# *Cladosporium species novum* Invasive Pulmonary Infection in a Patient with Post-COVID-19 Syndrome and AIDS

**DOI:** 10.3390/diagnostics15060781

**Published:** 2025-03-20

**Authors:** Milorad Bijelović, Nikola Gardić, Aleksandra Lovrenski, Danijela Petrović, Gordana Kozoderović, Vesna Lalošević, Vuk Vračar, Dušan Lalošević

**Affiliations:** 1 Thoracic Surgery Clinic, Institute for Pulmonary Diseases of Vojvodina, Put Doktora Goldmana 4, 21204 Novi Sad, Serbia; milorad.bijelovic@institut.rs; 2 Department of Surgery, Faculty of Medicine Foca, University of East Sarajevo, 73300 Foča, Bosnia and Herzegovina; 3 Department for Pathology and Molecular Diagnostics, Institute for Pulmonary Diseases of Vojvodina, Put Doktora Goldmana 4, 21204 Novi Sad, Serbia; aleksandra.lovrenski@mf.uns.ac.rs; 4 Faculty of Medicine, University of Novi Sad, Hajduk Veljkova 3, 21000 Novi Sad, Serbia; dusan.lalosevic@mf.uns.ac.rs; 5 Department of Natural Sciences and Management in Education, Faculty of Education in Sombor, University of Novi Sad, Podgorička 4, 25000 Sombor, Serbia; petrovid@tcd.ie (D.P.); gocakozoderovic@gmail.com (G.K.); 6 Department of Veterinary Medicine, Faculty of Agriculture, University of Novi Sad, Trg Dositeja Obradovića 8, 21000 Novi Sad, Serbia; vesna.lalosevic@polj.uns.ac.rs (V.L.); vuk.vracar@polj.uns.ac.rs (V.V.)

**Keywords:** *Cladosporium*, mycoses, lung, human, Serbia

## Abstract

**Background and Clinical Significance:** Since the prevalence of fungal lung infections is increasing, certain agents, such as *Cladosporium* spp., have emerged as unexpected causes. *Cladosporium* spp. fungi are ubiquitous in environments such as soil, fruits, and wine corks; they are a part of the normal human skin flora; and they are known respiratory allergens. **Case Presentation**: A patient with a history of post-COVID-19 syndrome and AIDS presented with lung pathology indicative of an invasive fungal infection. The initial histopathological examination revealed numerous yeast-like cells with narrow-based budding, which led to a mistaken diagnosis of cryptococcosis. However, further detailed examination revealed sparse hyphae in the lung tissue, suggesting a more complex fungal infection. Molecular analyses and sequence BLAST alignment were performed, ultimately identifying the infectious agent as “*Cladosporium species novum*”, a rare cause of invasive pulmonary cladosporiasis. **Conclusions**: Invasive pulmonary cladosporiasis is a rare condition, and the morphological features of the fungus alone were insufficient to establish a correct diagnosis. A comprehensive pathohistological and molecular approach with bioinformatics tools is essential for the correct identification of rare and potentially life-threatening fungal pathogens in immunocompromised patients.

## 1. Introduction

COVID-19 not only caused acute pulmonary disease, which can sometimes be fatal, but also led to post-COVID-19 syndrome, with fungal infections playing a significant role in morbidity. As a consequence of the COVID-19 pandemic, numerous cases of lung fungal co-infections have been reported, particularly cryptococcosis, in both immunocompetent and immunocompromised patients [1].

Since the prevalence of fungal lung infections is increasing, certain agents, such as *Cladosporium* spp., have emerged as unexpected causes. *Cladosporium* spp. fungi are ubiquitous in environments such as soil, fruits, and wine corks; they are a part of the normal human skin flora; and they are known respiratory allergens [2,3]. They have been associated with “salami brusher’s disease” [4] and may act as plant pathogens and contaminants of heritage materials. However, this fungus very rarely causes superficial invasive infections, such as keratitis [5].

Systemic infections in humans and animals caused by *Cladosporium* spp. are exceedingly rare [6]. A previous study reported a case of peritonitis caused by *Cladosporium* in a patient undergoing peritoneal dialysis [7]. Pulmonary phaeohyphomycosis caused by *Cladosporium* cladosporioides has been documented twice [8,9]. In contrast, numerous papers have reported the non-invasive respiratory manifestation of *Cladosporium* spp., mainly as an aeroallergen capable of causing sensitization and allergic fungal rhinosinusitis or bronchial asthma [10,11,12,13,14]. Although causing non-invasive mycoses, a recent study identified *Cladosporium* spp. as agents significantly associated with decreases in lung function in patients with asthma [15], which is in line with previous reports on the exacerbation of asthma symptoms and poor asthma management in those exposed to *Cladosporium* spp. [16,17].

A related fungus, *Cladophialophora arxii*, formerly classified under the genus *Cladosporium*, has been known to cause lung infections like chromoblastomycosis, albeit extremely rarely [18]. There are also documented cases of brain infections and abscess formation caused by *Cladophialophora bantiana* (formerly *Cladosporium bantianum*) [19]. A body of evidence of this type of invasive fungal infection elucidates a high mortality rate, particularly in immunosuppressed patients, due to late diagnosis and the absence of adequate medication [20,21]. Nonetheless, mortality due to this cystic brain infection can be reduced by including appropriate neurosurgery and powerful (triple) antifungal therapy [22,23]. According to the Advisory Committee on Dangerous Pathogens (ACDP) and the Japanese Society for Medical Mycology, *C. bantiana* is classified in the human pathogen hazard group 3 (risk group 3), as fungi with a high risk for individuals, since it can cause devastating infections in humans, and a low risk for the community, as it has a low potential for human-to-human transmission [24,25].

The emergence of rare fungal pathogens has become a growing concern in recent years, largely due to the increasing number of immunocompromised individuals. These infections are often severe, with high rates of morbidity and mortality, while their diagnosis and management present significant challenges. Prompt identification is critical but requires both clinical vigilance and reliable diagnostic tools. Although traditional methods such as direct microscopy, culture, and histopathology remain fundamental, the adoption of faster and more precise molecular techniques is necessary to enable early and targeted treatment [26,27]. This study presents a case of a patient with AIDS and post-COVID-19 syndrome who developed a pulmonary fungal infection caused by a *Cladosporium* species novum, established by histopathological examination, PCR molecular diagnostics, and bioinformatics analysis.

## 2. Case Report

### 2.1. Patient’s Medical History and Clinical Course of the Disease

A 56-year-old man from northern Serbia, with a 35-pack-year history of tobacco use and a medical history of hypertension, underwent cardiac stent placement in 2019 following a myocardial infarction. In 2021, he developed severe COVID-19 pneumonia, which was followed a year later by diffuse interstitial pneumonia. During a follow-up evaluation in 2023, radiological studies, including computed tomography, revealed bilateral apical pulmonary infiltrates with cavitation measuring 1.9 to 2.5 cm in diameter. On admission, initial laboratory findings were unremarkable (Table 1).

The patient underwent video-assisted thoracoscopic surgery (VATS) to address a peripheral lesion in the left lung. Intraoperatively, a whitish nodular lesion was identified in the S3 segment beneath the visceral pleura, and an atypical resection was performed using two endoscopic stapler loads, ensuring a protective margin of healthy tissue. The resected lung tissue was submitted for histopathological examination, which revealed necrotizing granulomatous inflammation on frozen section analysis. The patient was discharged in good general condition two days after the procedure. At the first follow-up visit, conducted six days later, local findings were unremarkable. Based on the initial clinical presentation and histopathological findings suggestive of tuberculosis, tuberculostatic therapy was initiated at the regional healthcare facility.

### 2.2. Histopathology

**Figure 1:**
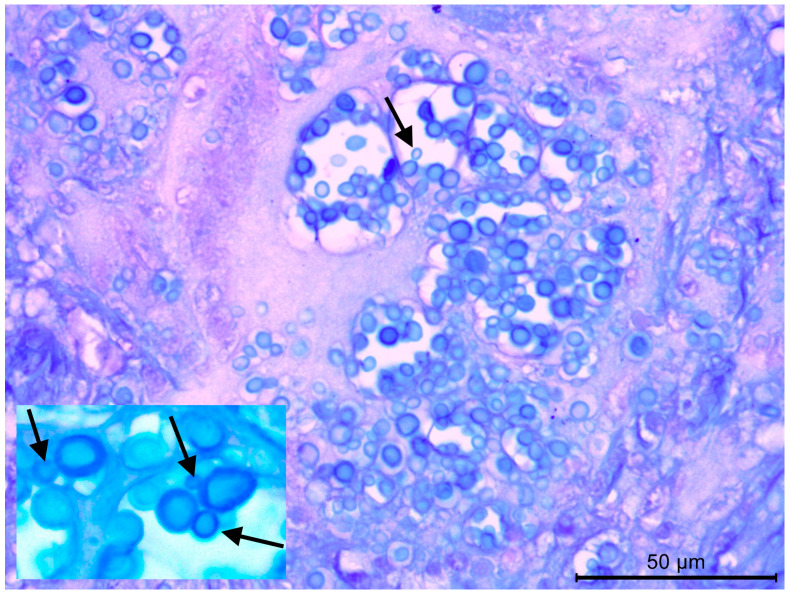
Lung tissue stained with Alcian–HE stain, highlighting numerous yeast-like cells within the cytoplasm of multinucleated giant cells. The yeast forms exhibit narrow-based budding (indicated by arrows), a characteristic feature of certain fungal infections. The organisms are distributed in clusters, with some appearing within vacuolated spaces. The inset provides a higher magnification view, emphasizing the morphology of individual yeast cells and their budding pattern. The background tissue demonstrates an inflammatory response with areas of necrosis.

**Figure 2:**
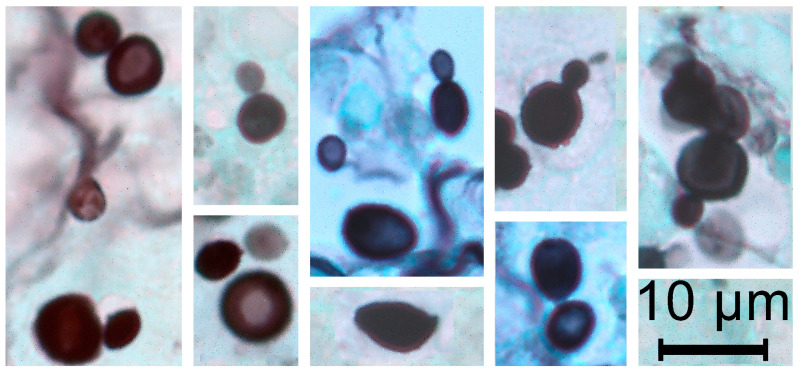
Grocott’s methenamine silver (GMS)-stained lung tissue biopsy, highlighting numerous yeast-like fungal cells. The fungal organisms appear dark brown to black against a pale green background, as expected of the GMS stain, which selectively stains fungal cell walls rich in polysaccharides. The variation in size and shape of the fungal elements is notable, with some appearing round and others slightly elongated.

The initial frozen section histopathological analysis revealed granulomatous necrotic inflammation. On the definitive histological slides, coalescent epithelioid granulomas with multinucleated giant cells were observed, containing numerous phagocyted and free fungal cells. Conventional staining techniques, including Hematoxylin–Eosin (HE), Alcian–HE, Periodic Acid–Schiff (PAS), and particularly Grocott Methenamine Silver (GMS) stain, demonstrated numerous yeast-like cells measuring 5 to 10 µm, occasionally exhibiting budding. Hyphal elements, however, were not readily identifiable, and Giemsa staining failed to reveal fungal cells. The yeast-like cells were disseminated within alveolar spaces and pulmonary cavities, sometimes forming aggregates or chain-like structures resembling fungal conidia. In these areas, the inflammatory infiltrates were abundant and predominantly composed of epithelioid and multinucleated giant cells. Notably, the cytoplasm of these cells contained large vacuoles densely packed with yeast-like cells (Figure 1 and Figure 2). Based on these findings, including the observation of yeast-like cells occasionally displaying narrow-based budding, a diagnosis of cryptococcosis was suggested.

**Figure 3:**
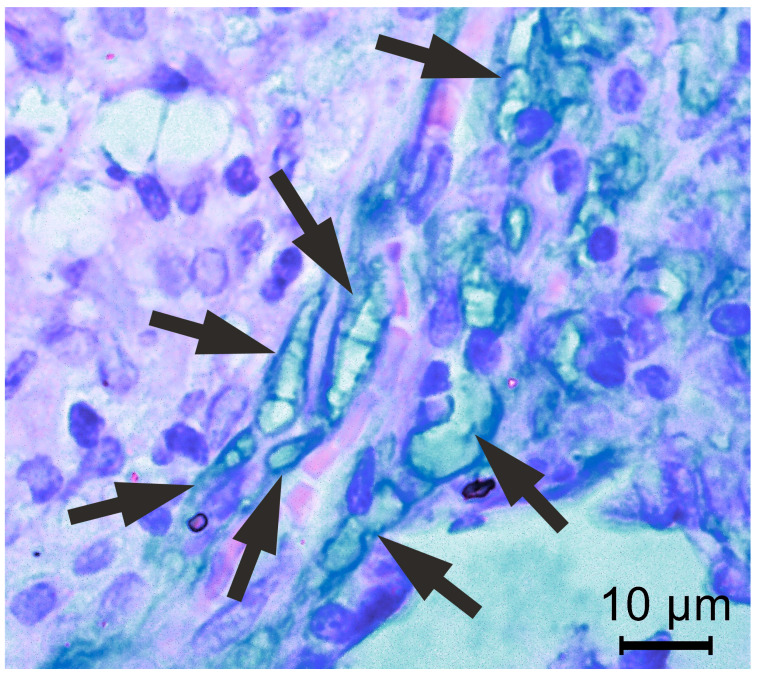
Lung tissue stained with Alcian–HE, highlighting septate fungal hyphae. In very few areas, the fungi appeared as convoluted, elongated hyphae with irregular lumen diameters (indicated by arrows). The surrounding lung tissue demonstrates an inflammatory response, with numerous immune cells infiltrating the area.

However, upon a meticulous examination of the slides, Alcian-positive irregular convoluted tubular structures were identified, indicating the presence of septate fungal hyphae (Figure 3).

### 2.3. Follow-Up with Progression of the Disease and Treatment

**Figure 4:**
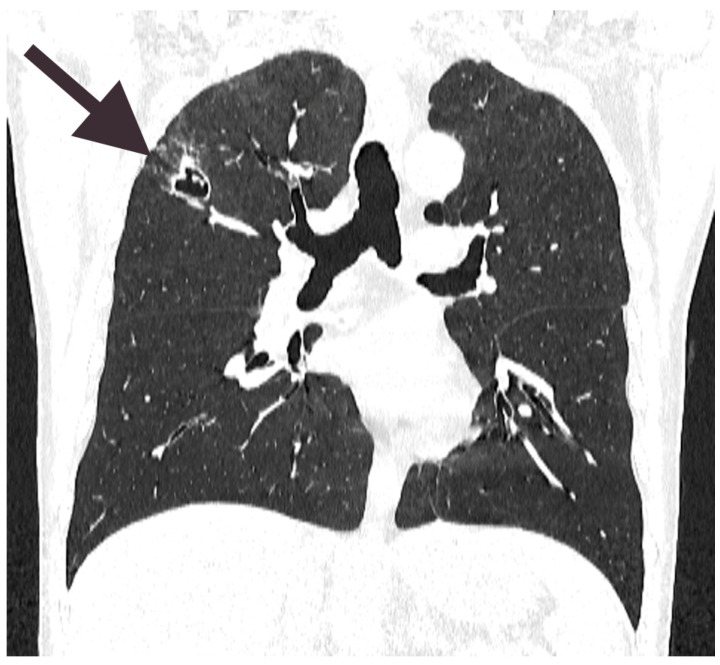
The scan reveals a cavitary lesion in the upper lobe of the right lung, as indicated by the arrow. The cavity appears as a well-defined radiolucent area with a thick, irregular wall and internal air content. The small hyperdense structure within the lumen of the cavitary lesion may represent a fungal ball. Surrounding lung parenchyma exhibits signs of consolidation and fibrotic changes.

Based on the findings of a yeast infection and the suspected diagnosis of cryptococcosis during the follow-up visit, antituberculosis therapy was discontinued, and antifungal treatment with fluconazole (200 mg, 3 mg/kg) was initiated in accordance with the recommendations of the Infectious Diseases Society of America. A follow-up chest CT scan (Figure 4) revealed the progression of the lesion in the right lung, which had increased from 19 mm to 25 mm in diameter and demonstrated the presence of a fungal ball adherent to the wall of the cavernous lesion. The patient developed a low-grade fever, with a maximum temperature of 37.6 °C. Given the lesion’s progression, surgical intervention was deemed necessary. Initial laboratory findings revealed leucopenia with a white blood cell count of 2.8 × 10^9^/L (reference range: 4.0–11.0 × 10^9^/L) and lymphopenia, with an absolute lymphocyte count of 0.35 × 10^9^/L (reference range: 1.0–4.5 × 10^9^/L), and a relative lymphocyte count of 12 (reference range: 20.0–46.0%) indicative of immunosuppression (Table 1). Due to the lesion’s deep location within the lung parenchyma, resection was performed using thermocautery along the lesion walls, with intraoperative identification by palpation to preserve surrounding healthy tissue. The resected specimen was sent for histopathological examination, which confirmed the fungal etiology and revealed findings consistent with the previously described organism’s morphology. The postoperative course was uneventful, and the patient was discharged in stable condition three days after the procedure.

### 2.4. DNA Extraction, PCR Amplification, and Sequencing

Total fungal DNA was extracted from the formalin-fixed paraffin-embedded (FFPE) lung tissue according to the manufacturer’s instructions (GeneMATRIX Plant & Fungi DNA Purification Kit, EURx Ltd., Gdansk, Poland). The conventional polymerase chain reaction (PCR) method was utilized to detect fungal DNA in FFPE tissue since the histopathology examination of the patient’s lung tissue samples pointed to a fungal infection. PCR was performed in order to target the internal transcribed spacer region (ITS2) and 5.8S rRNA gene, using a universal primer pair: ITS-3F (5′-GCATCGATGAAGAACGCAGC-3′) and ITS-4R (5′TCCTCCGCTTATTGATATGC-3′) [28] The total volume of each PCR was 25 μL composed of 12.5 μL of HotStart Taq2x MasterMix (BioLabs, Heidelberg, Germany), 0.25 μL of each primer (20 μM), 7 μL of nuclease-free water, and 5 μL of DNA extract. Running conditions were an initial denaturation at 94 °C for 5 min, followed by 40 cycles of denaturation at 94 °C for 60 s, annealing at 51 °C for 60 s and elongation at 72 °C for 60 s, and a final elongation at 72 °C for 10 min [29]. PCR products (length ~330 bp) were analyzed on a 2% agarose gel that was stained with ethidium bromide (1 µg/mL final concentration in ddH_2_O for 30 min) and visualized using UV gel documentation system SERVA Blue-Cube 300 (SERVA Electrophoresis GmbH, Heidelberg, Germany). A molecular weight marker (GeneRuler 100bp Plus DNA Ladder, Thermo Scientific, Thermo Fisher Scientific Inc., Waltham, MA, USA) was included in each run, while Saccharomyces cerevisiae was used as a positive control. PCR products in individual tubes were sent for sequencing and the results were obtained using the Standard-Seq method of Sanger sequencing technology (3730xl DNA Analyzer, Macrogen Europe, Amsterdam, The Netherlands).

### 2.5. Bioinformatics Approach and Alignments

BLAST 2.16.0 (the Basic Local Alignment Search Tool, https://blast.ncbi.nlm.nih.gov/Blast.cgi, accessed on 23 July 2024) was used to compare the obtained nucleotide DNA sequence (query) to the sequences deposited in the BLAST database (subject sequences). A text-based sequence file was used for the BLAST search, and the obtained sequence (query) was submitted to GeneBank and was provided with a GenBank accession number (PQ067362) (Table 2).

The BLAST yielded alignments with small Expect (E) values, indicating database matches of very high quality. The program algorithms generated the alignments and calculated their statistical significance. None of the matches disclosed a score of 100% identity. However, all displayed sequences had a great match with the query sequence ranging from 98 to 99%. The query coverage for all sequences was between 99 and 100%, indicating that the sequence in the database spans most of the query sequence or the complete query sequence. All hundred yielded alignments originating within the sequences of the eukaryotic, fungal, Ascomycota genus *Cladosporium*, with the exceptions of two described as uncultured fungal species. The most prominent matches that achieved the highest identity scores (99%) and ranged as the most possible organism for the origin of our query sequence are listed in Table 3.

**Figure 5:**
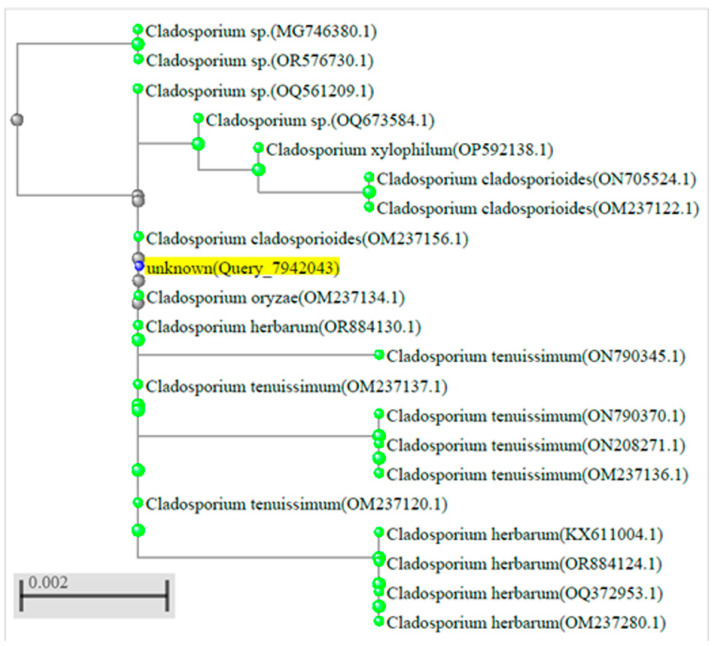
Phylogenetic tree. The tree is generated by the BLAST tool and based on the neighbor-joining method. The rectangle cladogram displays the phylogenetic relationship between our query sequence (unknown in yellow, blue nod) and 20 of the most homologous sequences in BLAST search (gray and green nods *Ascomycete fungi*). The tree highlights the closest relationship between the query sequence and two Ascomycetes: OM237156.1 and OM237134.1 (on 23 July 2024).

Taking into account the obtained BLAST results, we confirmed that DNA isolated from the patient’s FFPE tissue lung samples originated from *Cladosporium* spp. fungi. Further, phylogenetic analysis was performed using the BLAST tool (based on the Jukes–Cantor method), and the tree was built from the distance matrices using the neighbor-joining method. Phylogenetic analysis based on the partial nucleotide sequences of our query sequence and subject sequences (20 with the highest score) proposed the closest relationship of our query sequence with database sequences confirmed in two species: *Cladosporium cladosporioides* (OM237156.1) and *Cladosporium oryzae* (OM237134.1) (Figure 5).

### 2.6. Follow-Up

At the follow-up examination 10 days post-discharge, the patient was afebrile and in good general condition, with normal local findings and a chest X-ray consistent with the surgical interventions, showing no evidence of residual lung infection. However, the patient reported intermittent evening fevers, with temperatures reaching up to 38 °C. At the subsequent check-up seven days later, surgical findings remained unremarkable, but the patient continued to experience fevers of 38 °C. Given the persistence of fever despite complete surgical removal of the lung fungal infection, further investigation was initiated to explore potential underlying immunodeficiency or other causes of unexplained febrile conditions. Testing confirmed human immunodeficiency virus (HIV) infection at the regional healthcare institution, with only 100 cells/µL of CD4 lymphocytes. The patient was subsequently referred to the Infectious Diseases Clinic for specialized management. 

## 3. Discussion

Tuberculosis remains the most common opportunistic infection in immunosuppressed HIV patients; however, systemic fungal diseases are increasingly being recognized in this population [8,30]. The genus *Cladosporium* is primarily associated with allergic conditions such as asthma, but infections caused by this genus are exceedingly rare and almost exclusively reported in immunocompromised individuals [31]. Notably, in 2013, the first documented case of *Cladosporium cladosporioides* infection in an immunocompetent individual was reported. This patient, who had occupational exposure to the pathogen through cork processing, highlighted an unusual route of infection [32]. 

In contrast to fungal co-infections observed in patients with acute COVID-19—where the most commonly reported pathogens included *Candida* (7.1%), *Aspergillus* spp. (4.6%), *Mucor* spp. (1.3%), and *Cryptococcus* (0.5%) [33,34]—we found that fungal infections occurring in patients who had recovered from COVID-19 were predominantly caused by *Mucor* spp. and *P. jirovecii* [35]. A key risk factor for invasive fungal infections is corticosteroid use, which is frequently prescribed for severe COVID-19 cases due to its association with reduced mortality and a decreased need for mechanical ventilation. However, corticosteroids are known to have immunosuppressive effects by inhibiting macrophage differentiation and cytokine production. Their use in COVID-19 treatment has been shown to increase the risk of invasive fungal infections by 3.33 times compared to patients who do not receive corticosteroids [36]. In the literature, we did not find a case of *Cladosporium* lung infection in a patient who had a history of COVID-19. Considering the scarcity in the literature of such cases, there is no clear consensus in management, so the treatment should remain conventional. 

In pulmonary pathology, certain fungal infections, such as aspergillosis or mucormycosis, are often straightforward to recognize. However, identifying yeast-like organisms can be challenging and may be nearly impossible based on morphological criteria alone. In the histopathological differential diagnosis for this case, several yeast-like fungi were considered, including Saccharomyces (associated with “baker’s lung nodules”) [37]. Initially, the infectious fungus was erroneously identified as *Cryptococcus neoformans*.

The subsequent discovery of hyphal elements in the Alcian–HE-stained lung tissue biopsy suggested the presence of a dimorphic fungus. In this patient’s lung biopsy, fungal organisms were exceptionally numerous. Ultimately, sequencing and bioinformatics analysis unequivocally confirmed the presence of a single fungal genus, *Cladosporium*.

Fungal DNA extraction from FFPE material proved to be an exceptionally challenging task, consistent with previously reported obstacles, including low DNA concentrations, potential degradation, and the presence of various inhibitors [38]. Moreover, the PCR detection of fungi exhibits significantly lower sensitivity when fungal DNA is derived from FFPE tissue samples. These factors collectively contributed to the difficulties in achieving a molecular confirmation of *Cladosporium* spp. [26].

Interestingly, Batra et al. described a brain abscess in which *Cladosporium sphaerospermum* was isolated. However, in histological slides stained with GMS, many round spores (likely yeast-like conidia) observed in 2D images were misidentified, as the authors stated “no fungal profiles seen” [39]. In this case, yeast-like forms predominated in the tissue samples, similar to our case, but without clearly visible hyphal structures. In contrast, in our patient’s samples, recognizable hyphal elements were observed exclusively in Alcian staining, which corresponds to the dimorphic nature of *Cladosporium fungi*.

*Cladosporium* is a large genus of dimorphic fungi comprising 169 species, of which only a few are true human pathogens. Some species, such as the *Cladosporium sphaerospermum*, produce oval or ellipsoidal yeast-like spores or conidia, which have been identified in human tissue. Approximately 40% of clinical isolates of *Cladosporium* spp. represent previously uncharacterized species [6]. In our case, we hypothesized that our isolate might be a new species, as no identical sequence was found in the BLAST database. However, further molecular analysis and proper taxonomic revision are required to definitively confirm this. This finding also holds significant implications for understanding the potential causes of lung fungal infections and the need for accurate diagnostic methods.

The patient continued HIV treatment at another hospital, and therefore, detailed medical records regarding their HIV management were unavailable. This lack of information represents a limitation of our study, as it may have influenced the clinical course and outcomes observed in our case. Consequently, a more comprehensive assessment of potential interactions between HIV treatment and the studied condition was not possible.

## Figures and Tables

**Table 1:** Patient’s complete blood count prior to first and second surgery.

Parameter	Before 1st Surgery	Before 2nd Surgery	Reference Range
RBC	4.65 × 10^12^/L	4.24 × 10^12^/L	3.8–5.63 × 10^12^/L
Hgb	134 g/L	126 g/L	125–175 g/L
Hct	0.408 1/L	0.380 1/L	0.367–0.53 1/L
MCV	88 fL	90 fL	80–100 fL
MCH	28.7 pg	29.7 pg	27.4–33.9 pg
MCHC	328 g/L	332 g/L	320–360 g/L
WBC	4.2 × 10^9^/L	2.8 × 10^9^/L	4–11 × 10^9^/L
Neu %	51%	75%	44–75%
Neu #	2.11 × 10^9^/L	2.14 × 10^9^/L	2.0–7.6 × 10^9^/L
Lym %	34%	12%	20–46%
Lym #	1.4 × 10^9^/L	0.35 × 10^9^/L	1.0–4.5 × 10^9^/L
Mon %	12%	12%	2–12%
Mon #	0.48 × 10^9^/L	0.33 × 10^9^/L	0.2–0.8 × 10^9^/L
Eos %	3.4%	0.3%	0.1–7%
Eos #	0.14 × 10^9^/L	0.01 × 10^9^/L	0.1–0.6 × 10^9^/L
Bas %	0.6%	0.4%	0.2–2%
Bas #	0.02 × 10^9^/L	0.01 × 10^9^/L	0.0–0.20 × 10^9^/L
Plt	239 × 10^9^/L	190 × 10^9^/L	150–450 × 10^9^/L

Abbreviations: RBC—red blood cells; Hgb—hemoglobin; Hct—hematocrit; MCV—mean corpuscular volume; MCH—mean corpuscular hemoglobin; MCHC—mean corpuscular hemoglobin concentration; WBC—white blood cells; Neu—neutrophils; Lym—lymphocytes; Mon—monocytes; Eos—eosinophils; Bas—basophils; Plt—platelets; %—relative value; #—absolute count.

**Table 2:** GenBank accession number and main characteristics associated with query sequence.

Sequence ID	Organism	Host	Isolation Source	Location	Accession Number
C1	*Cladosporium* spp.	*Homo sapiens*	Lungs	Serbia	PQ067362

**Table 3:** The most prominent scores according to the BLAST search results (on 23 July 2024).

No	Organism	Query Cover	E-Value	Percentage Identity	No
1.	*Cladosporium herbarum*, isolate ZS9–10	100%	1 × 10^−147^	98.68%	OR884130.1
2.	*Cladosporium tenuissimum*, isolate TJU_JAN24	100%	1 × 10^−147^	98.68%	OM237137.1
3.	*Cladosporium tenuissimum*, isolate TJU_JAN7	100%	1 × 10^−147^	98.68%	OM237120.1
4.	*Cladosporium cladosporioides*, isolate TJU_JAN44	99%	5 × 10^−147^	98.68%	OM237156.1
5.	*Cladosporium oryzae*, isolate TJU_JAN21	99%	5 × 10^−147^	98.68%	OM237134.1

## Data Availability

The original contributions presented in this study are included in the article. Further inquiries can be directed to the corresponding author.

## References

[1] Chan K.S., Lai C.C., Yu W.L., Chao C.M. (2022). COVID-19 Associated with Cryptococcosis: A New Challenge during the Pandemic. JoF.

[2] Agarwal R., Sehgal I.S., Muthu V., Denning D.W., Chakrabarti A., Soundappan K., Garg M., Rudramurthy S.M., Dhooria S., Armstrong-James D. (2024). Revised ISHAM-ABPA working group clinical practice guidelines for diagnosing, classifying and treating allergic bronchopulmonary aspergillosis/mycoses. Eur. Respir. J..

[3] Chinn S., Burney P., Sunyer J., Jarvis D., Luczynska C., On Behalf Of The European Community Respiratory Health Survey (1999). Sensitization to individual allergens and bronchial responsiveness in the ECRHS. Eur. Respir. J..

[4] Marvisi M., Balzarini L., Mancini C., Mouzakiti P. (2015). A new type of Hypersensitivity Pneumonitis: Salami brusherâ€^TM^s disease. Monaldi Arch. Chest Dis..

[5] Xuan R., Hong S.C., Trinh T., Coroneo M.T., Petsoglou C. (2023). Case Series of Rare Fungal Keratitides: Experiences from a Quaternary Eye Hospital in Sydney, Australia. JoF.

[6] Sandoval-Denis M., Gené J., Sutton D.A., Wiederhold N.P., Cano-Lira J.F., Guarro J. (2016). New species of *Cladosporium* associated with human and animal infections. Pers.-Int. Mycol. J..

[7] Tasic S., Miladinovic-Tasic N. (2007). *Cladosporium* spp.—Cause of opportunistic mycoses. Acta Fac. Medicae Naissensis.

[8] Castro A.S., Oliveira A., Lopes V. (2013). Pulmonary phaeohyphomycosis: A challenge to the clinician. Eur. Respir. Rev..

[9] Kaneda M., Nagaoka K., Kawasuji H., Matsunaga K., Inomata M., Miyazaki Y., Nakashima A., Yamamoto Y. (2023). Pulmonary abscess caused by *Cladosporium* cladosporioides after receiving outpatient chemotherapy. J. Infect. Chemother..

[10] Vacher G., Niculita-Hirzel H., Roger T. (2015). Immune responses to airborne fungi and non-invasive airway diseases. Semin. Immunopathol..

[11] Denning D.W., Chakrabarti A. (2017). Pulmonary and sinus fungal diseases in non-immunocompromised patients. Lancet Infect. Dis..

[12] Hughes K.M., Price D., Torriero A.A.J., Symonds M.R.E., Suphioglu C. (2022). Impact of Fungal Spores on Asthma Prevalence and Hospitalization. Int. J. Mol. Sci..

[13] Nasiri-Jahrodi A., Sheikholeslami F.M., Barati M. (2023). *Cladosporium* tenuissimum-induced sinusitis in a woman with immune-deficiency disorder. Braz. J. Microbiol..

[14] Oliveira M., Oliveira D., Lisboa C., Boechat J., Delgado L. (2023). Clinical Manifestations of Human Exposure to Fungi. JoF.

[15] Xu X., Ding F., Hu X., Yang F., Zhang T., Dong J., Xue Y., Liu T., Wang J., Jin Q. (2023). Upper respiratory tract mycobiome alterations in different kinds of pulmonary disease. Front. Microbiol..

[16] Denning D.W., O’Driscoll B.R., Hogaboam C.M., Bowyer P., Niven R.M. (2006). The link between fungi and severe asthma: A summary of the evidence. Eur. Respir. J..

[17] Sharpe R.A., Bearman N., Thornton C.R., Husk K., Osborne N.J. (2015). Indoor fungal diversity and asthma: A meta-analysis and systematic review of risk factors. J. Allergy Clin. Immunol..

[18] Obeagu E.I., Onuoha E.C. (2023). Tuberculosis among HIV patients: A review of Prevalence and Associated Factors. Int. J. Adv. Res. Biol. Sci..

[19] Brown G.D., Denning D.W., Gow N.A.R., Levitz S.M., Netea M.G., White T.C. (2012). Hidden Killers: Human Fungal Infections. Sci. Transl. Med..

[20] Levin T.P., Baty D.E., Fekete T., Truant A.L., Suh B. (2004). *Cladophialophora bantiana* Brain Abscess in a Solid-Organ Transplant Recipient: Case Report and Review of the Literature. J. Clin. Microbiol..

[21] Chakrabarti A., Kaur H., Rudramurthy S.M., Appannanavar S.B., Patel A., Mukherjee K.K., Ghosh A., Ray U. (2016). Brain abscess due to Cladophialophora bantiana: A review of 124 cases. Med. Mycol..

[22] Garzoni C., Markham L., Bijlenga P., Garbino J. (2008). *Cladophialophora bantiana*: A rare cause of fungal brain abscess. Clinical aspects and new therapeutic options. Med. Mycol..

[23] Lortholary O., Garcia-Hermoso D., Sturny-Leclère A., Sitbon K., Nourrisson C., Letscher-Bru V., Desbois-Nogard N., Bani-Sadr F., Bastides F., Bienvenu B. (2024). Reappraising Cladophialophora bantiana phaeohyphomycosis in France: Retrospective nation-based study. Lancet Microbe.

[24] The Advisory Committee on Dangerous Pathogens (ACDP) (2023). The Approved List of Biological Agents.

[25] Tashiro M., Takazono T., Izumikawa K. (2024). Overview of Fungi Classified as Risk Group 3 by the Japanese Society for Medical Mycology. Med. Mycol. J..

[26] Hien H.T.A., Thanh T.T., Thu N.T.M., Nguyen A., Thanh N.T., Lan N.P.H., Simmons C., Shikuma C., Chau N.V.V., Thwaites G. (2016). Development and evaluation of a real-time polymerase chain reaction assay for the rapid detection of *Talaromyces marneffei MP1* gene in human plasma. Mycoses.

[27] Skiada A., Pavleas I., Drogari-Apiranthitou M. (2017). Rare fungal infectious agents: A lurking enemy. F1000Research.

[28] White T.J., Bruns T., Lee S., Taylor J., Sninsky J., White T. (1989). Amplification and Direct Sequencing of Fungal Ribosomal Rna Genes for Phylogenetics. PCR Protocols: A Guide to Methods and Applications.

[29] Muñoz-Cadavid C., Rudd S., Zaki S.R., Patel M., Moser S.A., Brandt M.E., Gómez B.L. (2010). Improving Molecular Detection of Fungal DNA in Formalin-Fixed Paraffin-Embedded Tissues: Comparison of Five Tissue DNA Extraction Methods Using Panfungal PCR. J. Clin. Microbiol..

[30] Wang R.J., Miller R.F., Huang L. (2017). Approach to Fungal Infections in Human Immunodeficiency Virus–Infected Individuals. Clin. Chest Med..

[31] Brischetto A., Kidd S., Baird R. (2015). First Reported Australian Case of Cladophilophora arxii: Features Consistent with Possible Primary Pulmonary Chromoblastomycosis. Am. Soc. Trop. Med. Hyg..

[32] Binford C.H., Dooley J.R. (1976). Diseases caused by fungi and actinomycetes-Deep mycoses. Pathology of Tropical and Extraordinary Diseases.

[33] Suleiman A.S., Islam M.A., Akter M.S., Amin M.R., Werkneh A.A., Bhattacharya P. (2023). A meta-meta-analysis of co-infection, secondary infections, and antimicrobial resistance in COVID-19 patients. J. Infect. Public Health.

[34] Scendoni R., Bury E., Ribeiro I.L.A., Cingolani M., Cameriere R., De Benedictis A., De Micco F. (2023). Leading Pathogens Involved in Co-Infection and Super-Infection with COVID-19: Forensic Medicine Considerations after a Systematic Review and Meta-Analysis. Pathogens.

[35] Ulloque-Badaracco J.R., Copaja-Corzo C., Hernandez-Bustamante E.A., Cabrera-Guzmán J.C., Huayta-Cortez M.A., Carballo-Tello X.L., Seminario-Amez R.A., Hueda-Zavaleta M., Benites-Zapata V.A. (2024). Fungal infections in patients after recovering from COVID-19: A systematic review. Ther. Adv. Infect..

[36] De León-Borrás R., DelPilar-Morales E., Rivera-Pérez N., Pallens-Feliciano M., Tirado-Gómez M., González-Sepúlveda L., Bertrán-Pasarell J. (2017). Factors Associated to Invasive Fungal Infection in Hispanic Patients with Hematological Malignancies. Bol. Asoc. Med. P. R..

[37] Ren P., Sridhar S., Chaturvedi V. (2004). Use of Paraffin-Embedded Tissue for Identification of *Saccharomyces cerevisiae* in a Baker’s Lung Nodule by Fungal PCR and Nucleotide Sequencing. J. Clin. Microbiol..

[38] Guarner J., Brandt M.E. (2011). Histopathologic Diagnosis of Fungal Infections in the 21st Century. Clin. Microbiol. Rev..

[39] Batra N., Kaur H., Mohindra S., Singh S., Shamanth A.S., Rudramurthy S.M. (2019). *Cladosporium* sphaerospermum causing brain abscess, a saprophyte turning pathogen: Case and review of published reports. J. Mycol. Méd..

